# Protease‐mediated maturation of M‐PMV reverse transcriptase into a functional heterodimer

**DOI:** 10.1002/pro.70469

**Published:** 2026-01-20

**Authors:** Marina Kapisheva, Petra Junková, Ondřej Vaněk, Zuzana Jalovcová, Ivana Křížová, Alžběta Dostálková, Michaela Rumlová

**Affiliations:** ^1^ Department of Biotechnology University of Chemistry and Technology Prague Czech Republic; ^2^ Institute of Organic Chemistry and Biochemistry, Czech Academy of Sciences Prague Czech Republic; ^3^ Department of Biochemistry Faculty of Science, Charles University Prague Czech Republic

**Keywords:** analytical ultracentrifugation, Mason‐Pfizer monkey virus, polymerase activity, proteolytic processing, reverse transcriptase

## Abstract

Reverse transcriptase (RT) of retroviruses orchestrates viral replication, yet its structural diversity remains poorly understood. Well‐studied RTs, such as those from HIV‐1, murine leukemia virus, and avian myeloblastosis virus, were characterized decades ago, but less prominent retroviruses have escaped detailed analysis. Despite being discovered alongside HIV‐1, the RT of Mason‐Pfizer monkey virus (M‐PMV) has resisted recombinant expression, leaving its properties unresolved. Here, we report the first detailed analysis of M‐PMV RT, a betaretroviral enzyme previously thought challenging to obtain recombinantly. Using baculovirus‐based expression in insect cells, we produced soluble full‐length RT that, upon proteolytic maturation by the M‐PMV protease, yielded a heterodimer composed of p65 and p51 subunits. Mass spectrometry, N‐terminal sequencing, and analytical ultracentrifugation demonstrated that full‐length RT forms a homodimer, which converts into a stable and more enzymatically active heterodimer following proteolytic removal of the C‐terminal RNase H domain from one subunit. Functional assays revealed that heterodimer formation enhances polymerase activity while preserving RNase H function, directly linking proteolytic maturation to enzymatic activation. Notably, this heterodimeric architecture is uncommon among betaretroviruses and resembles the well‐characterized lentiviral HIV‐1 RT. These results broaden the evolutionary perspective on RT heterodimerisation by revealing that this architecture extends into betaretroviruses.

## 1 INTRODUCTION

Retroviruses replicate by converting their single‐stranded RNA genome into a stably integrated DNA copy, a process catalyzed by viral reverse transcriptase (RT). This multifunctional RNA‐dependent DNA polymerase synthesizes complementary DNA and degrades the original RNA template (Huber et al., 2023). Discovered more than 50 years ago (Baltimore, 1970; Temin & Mizutani, 1970), RT paved the way for new experimental approaches and has become an essential tool in molecular biology, facilitating cDNA synthesis and transcriptome analysis. Despite this long‐standing interest, some reverse transcriptases are still not fully characterized, and the structural and biochemical diversity of RTs across retroviral genera remains poorly understood. In particular, the reverse transcriptase of Mason‐Pfizer monkey virus (M‐PMV), a betaretrovirus, has resisted recombinant production, and its structural organization and enzymatic characterization are largely unexplained. Here, we address this gap by combining recombinant expression with biochemical and biophysical approaches to define the structural and functional properties of M‐PMV RT.

In all seven genera of retroviruses: Alpharetrovirus, Betaretrovirus, Gammaretrovirus, Deltaretrovirus, Epsilonretrovirus, Spumavirus, and Lentivirus (Coffin et al., 1997), the reverse transcriptase is encoded within the *pol* gene and expressed as part of a Gag‐Pol polyprotein precursor through ribosomal frameshifting. The details of this strategy differ among retroviruses. In lentiviruses such as HIV‐1, a single frameshift produces Gag and Gag‐Pol polyproteins (Menéndez‐Arias et al., 2017), whereas the betaretrovirus M‐PMV employs two ribosomal frameshifts: the first near the 3′ end of *gag* yields Gag‐Pro and the second near the 3′ end of *pro* generates Gag‐Pro‐Pol (Marx, 2008). RT is released from these precursors by the viral protease during viral maturation. A direct consequence of this dual frameshift mechanism is that M‐PMV produces significantly lower levels of RT, roughly 10‐fold less than HIV‐1 (Kohoutová et al., 2009). Such limited expression suggests that M‐PMV RT may require intrinsically higher enzymatic activity to ensure efficient reverse transcription.

Despite their sequence diversity, all studied retroviral RTs share a conserved modular architecture consisting of two spatially separated domains: the DNA polymerase domain linked to the C‐terminal RNase H domain. The DNA polymerase domain forms a structure resembling a right‐hand conformation comprising the fingers, palm, and thumb subdomains, which form the nucleic acid‐binding cleft (Hizi & Herschhorn, 2008). The palm contains the catalytic site of the polymerase, which is essential for DNA synthesis and is formed by highly conserved amino acid residues. The fingers guide incoming nucleotides toward the template strand, while the thumb stabilizes the DNA/RNA hybrid during elongation. The connection subdomain links the polymerase core to the C‐terminal RNase H domain. The RNase H domain is responsible for degrading the RNA strand of RNA/DNA hybrids. Structurally, it adopts a fold of five mixed β‐sheets surrounded by four α‐helices. The catalytic center includes conserved acidic residues (Asp, Glu) that coordinate Mg^2+^ ions essential for cleavage (Herschhorn & Hizi, 2010).

Despite this conserved framework, RTs exhibit striking variability in their quaternary structure, as illustrated by the presence of either monomeric or dimeric organization. Lentiviral HIV‐1 RT, for example, is a well‐characterized RT heterodimer composed of p66 and p51 subunits (London, 2016; London, 2019; Zheng et al., 2015). The p66 subunit contains the active sites for both DNA polymerase and RNase H, whereas the p51 subunit primarily serves as a structural scaffold (Xavier Ruiz & Arnold, 2020). A similar heterodimeric organization is found in alpharetroviral RTs, such as avian leukosis virus (ALV). The larger 94 kDa β‐subunit of ALV RT contains polymerase, RNase H, and integrase domains; the smaller 63 kDa α‐subunit lacks the integrase domain (Hizi & Herschhorn, 2008; Werner & Wöhrl, 1999). By contrast, RTs from deltaretroviruses (e.g., bovine leukemia virus, BLV) and gammaretroviruses (e.g., murine leukemia virus, MLV) function as monomers (Das & Georgiadis, 2004; Perach & Hizi, 1999). Also, betaretroviral mouse mammary tumor virus (MMTV) RT has been characterized as a monomer (Taube et al., 1998). In contrast, the structural characterization of M‐PMV RT, another betaretroviral enzyme, remains limited.

The main obstacle to the detailed structural and biochemical characterization of M‐PMV RT has been the failure of previous attempts to produce the enzyme in bacterial systems using recombinant techniques. M‐PMV RT characterization has therefore been limited to measurement of its polymerase activity (Křízová et al., 2012). More recently, however, we have overcome the RT production problems and generated a specific anti‐M‐PMV RT antibody that enabled the detection of partially processed RT within virions (Dostálková et al., 2024). The detection of two distinct RT‐derived proteins in mature M‐PMV virions suggested that, in contrast to the monomeric RT of related betaretrovirus MMTV, M‐PMV may employ a different mode of organization. In this study, we address this issue by establishing a recombinant expression system for M‐PMV RT and applying complementary biochemical approaches to characterize its organization and activity. Our results demonstrate that, despite its classification within betaretroviruses, which otherwise contain predominantly monomeric RTs, M‐PMV RT undergoes protease‐mediated maturation to form a heterodimeric enzyme. This unexpected finding reveals that the structural and functional properties of M‐PMV RT more closely resemble those of HIV‐1 RT.

## 2 RESULTS

### 2.1 Sequence comparison of retroviral reverse transcriptases

**FIGURE 1:**
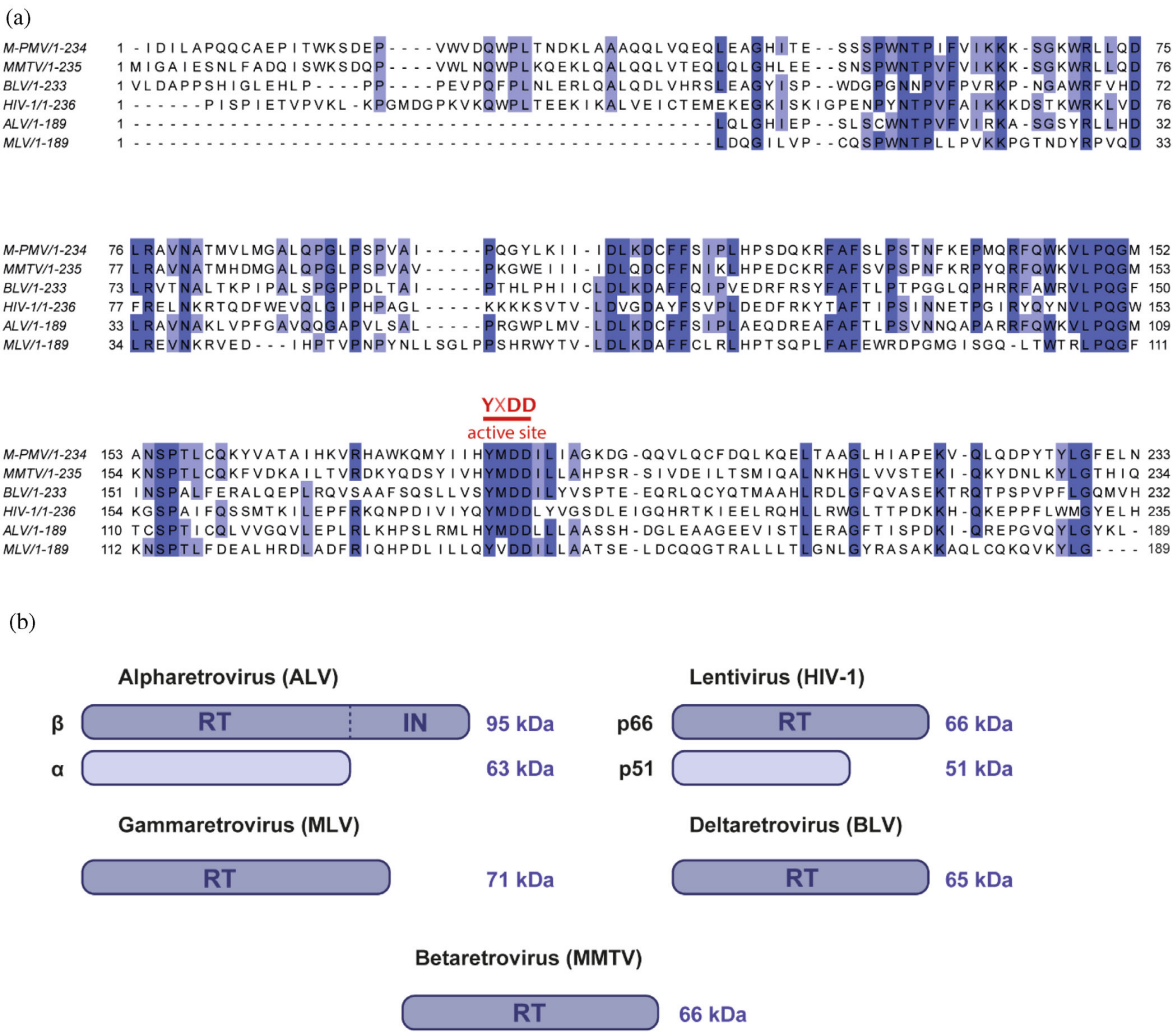
Comparative sequence alignment and structural representation of reverse transcriptases from different retroviral genera. (a) Partial sequence alignment of the fingers and palm subdomains from M‐PMV, MMTV, BLV, HIV‐1, ALV, and MLV RTs. Conserved regions are highlighted in blue and the canonical YXDD active‐site motif in red. The residues were numbered according to their UniProt sequences. Pairwise sequence identity comparisons were as follows: M‐PMV/MMTV: 62.4%; ALV: 48.2%; BLV: 37.2%; HIV‐1: 32.4%; MLV: 32.1%. (b) Schematic representation of RT subunit composition and approximate molecular masses across retroviral genera. Lentiviral RTs (e.g., HIV‐1) and alpharetroviral RTs (e.g., ALV) form heterodimers (p66/p51 and α/β, respectively), whereas gammaretroviral (MLV), deltaretroviral (BLV), and betaretroviral (MMTV) RTs are predominantly monomeric.

Reverse transcriptases across different retroviral genera share conserved features, retaining the structural motifs essential for their catalytic function, yet exhibit notable differences in both sequence and structural organization. A multiple sequence alignment of the fingers and palm subdomains from six representative RTs, M‐PMV and MMTV (Betaretrovirus), BLV (Deltaretrovirus), HIV‐1 (Lentivirus), ALV (Alpharetrovirus), and MLV (Gammaretrovirus) revealed several conserved regions (Figure 1a, blue). Among them, the canonical YXDD motif (highlighted in red) is a highly conserved sequence within the polymerase active site critical for catalytic activity. Pairwise sequence comparisons showed that M‐PMV RT is most related to MMTV RT (with 62.4% sequence identity), consistent with their close taxonomic placement within the betaretrovirus genus. Moderate similarity was observed with ALV (48.2%) and BLV (37.2%) RTs, while the lowest identity was observed with the more distantly related RTs of HIV‐1 (32.4%) and MLV (32.1%).

Despite these conserved elements, retroviral RTs exhibit distinct structural organization (Figure 1b). Alpharetroviral RTs (e.g., ALV) form heterodimers, with a 95 kDa β‐subunit that includes the polymerase, RNase H, and integrase domains and a 63 kDa α‐subunit lacking the integrase. Lentiviral RTs (e.g., HIV‐1) also form heterodimers, composed of a 66 kDa subunit (p66) containing both polymerase and RNase H domains and a 51 kDa subunit (p51) lacking the RNase H domain. In contrast, RTs characterized from gammaretroviruses (MLV, 71 kDa), deltaretroviruses (BLV, 65 kDa), and betaretroviruses (MMTV, 66 kDa) are monomeric.

### 2.2 Purification and proteolytic processing of recombinant M‐PMV reverse transcriptase

**FIGURE 2:**
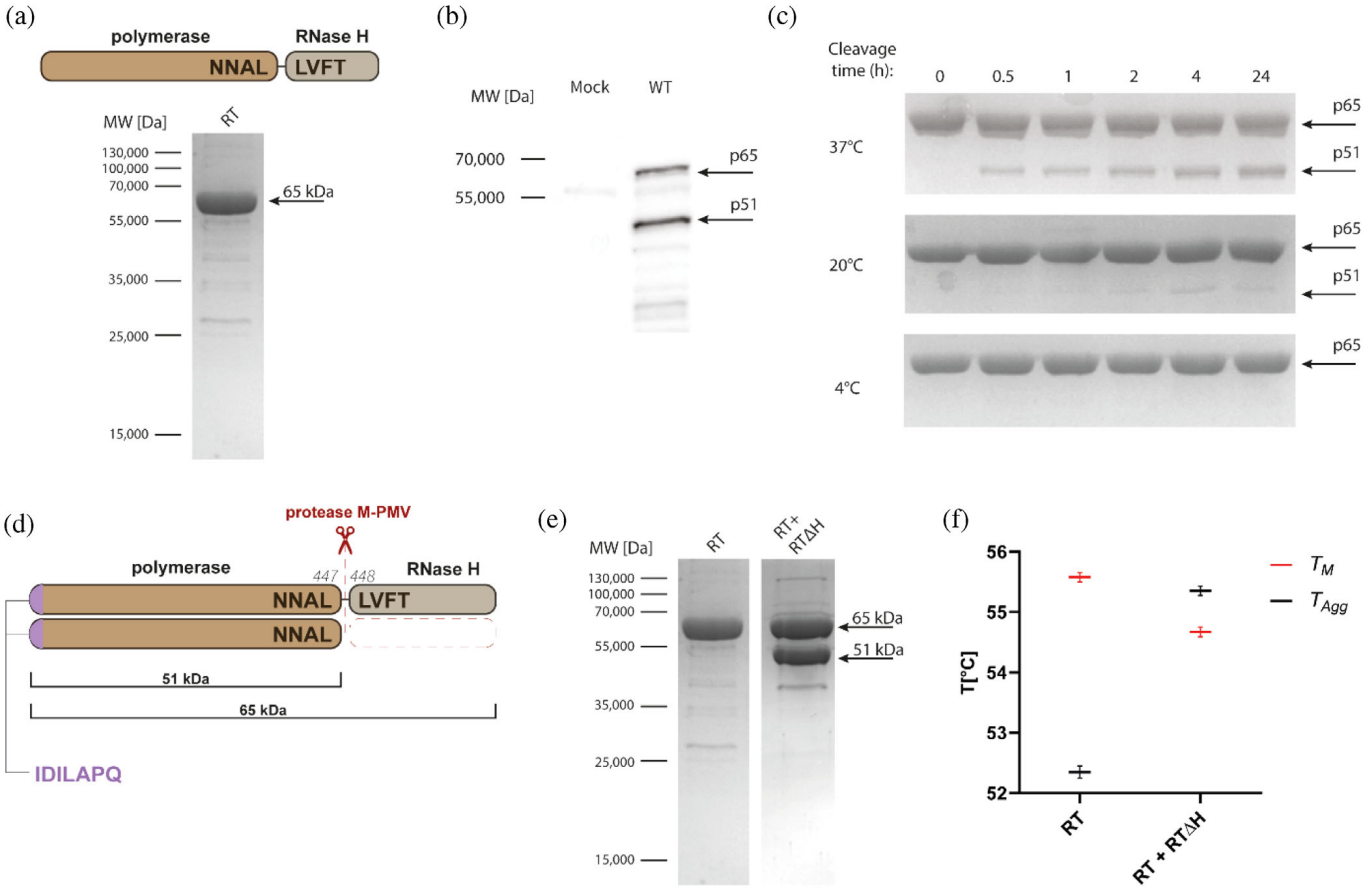
Purification and proteolytic processing of recombinant M‐PMV reverse transcriptase. (a) Upper panel: Schematic representation of M‐PMV RT, consisting of a polymerase domain and an RNase H domain connected by a protease cleavage site (NNAL↓LVFT). Lower panel: SDS‐PAGE analysis of purified recombinant full‐length M‐PMV RT. (b) Western blot detection of RT in virions collected from HEK293 cells transfected with the pSARM vector for 48 h using an RT‐specific polyclonal antibody. Two distinct bands corresponding to p65 and p51 were observed. (c) Proteolytic processing of purified RT by the viral PR13 protease. Efficient cleavage at 37°C generated two products: The full‐length p65 subunit (65 kDa) and the truncated p51 subunit (51 kDa). (d) Schematic representation of M‐PMV RT processing at the NNAL↓LVFT site by the viral protease, as determined by N‐terminal sequencing and ESI‐MS. Following cleavage, the major products were 64,875 Da (p65) and 51,209 Da (p51), both of which share an identical N‐terminal sequence IDILAPQ, shown in purple. (e) Side‐by‐side SDS‐PAGE analysis of both purified proteins. Left lane: Full‐length RT (His‐TEV‐RT‐TEV‐MBP). Right lane: Co‐purified full‐length RT with truncated RTΔH co‐expressed in Sf9 cells. (f) Comparison of the thermal stability parameters of RT and the RTΔH, showing melting temperatures (*T*_*M*_, red) and aggregation onset temperatures (*T*_Agg_, black). *T*_*M*_ was determined by nanoDSF as the inflection point of the 330/350 nm fluorescence ratio curve. *T*_Agg_ was obtained from turbidimetric measurements and corresponds to the temperature at which detectable protein aggregation begins. Data represent three independent measurements performed in duplicate for each sample and analyzed using Panta software.

Initial attempts to express M‐PMV RT recombinantly in *E. coli* were unsuccessful. Despite testing multiple bacterial strains, codon‐optimized RT constructs and variants with modified N‐ and C‐termini, none of these approaches yielded a soluble full‐length protein. These limitations prompted us to use the Bac‐to‐Bac baculovirus expression system. The gene encoding full‐length M‐PMV RT (Figure 2a, upper panel) was inserted into the pFASTBac vector and expressed in Sf9 insect cells. Recombinant RT was then purified using immobilized metal affinity chromatography (IMAC), followed by size‐exclusion chromatography. To enhance solubility, the full‐length construct contained C‐ and N‐terminal tags for maltose‐binding protein (MBP) and 6 × His, respectively, each separated from RT by a TEV cleavage site (His‐TEV‐RT‐TEV‐MBP). The solubility and stability of RT were also improved by the addition of trehalose to all purification buffers. In the absence of trehalose, the binding of His‐TEV‐RT‐TEV‐MBP to the HisTrap HP column was insufficient, likely due to steric hindrance of the His‐tag.

Approximately 1 mg of purified full‐length RT with >90% purity was obtained from a 500 mL Sf9 suspension culture, as confirmed by SDS‐PAGE and Coomassie staining (Figure 2a, lower panel). The purified full‐length 65 kDa RT was subsequently used to produce a polyclonal guinea pig antibody, which enabled RT detection in M‐PMV virions. Western blot analysis of virions produced in HEK293 cells revealed two distinct RT‐specific bands at 65 kDa (p65) and 51 kDa (p51) (Figure 2b). While the full‐length product was expected, the presence of a smaller band was unanticipated. This difference indicated the proteolytic removal of the 14 kDa RNase H domain.

To test proteolytic processing directly, purified full‐length RT was incubated with M‐PMV PR13 protease at 4°C, 20°C, and 37°C for time intervals ranging from 30 min to 24 h (Figure 2c). No cleavage was observed at 4°C, and only faint cleavage products appeared at 20°C, even after prolonged incubation at this temperature. In contrast, incubation at 37°C resulted in efficient proteolysis, with two distinct bands corresponding to p65 and truncated p51. After 24 h, both products accumulated to similar levels.

Subunit characterization was performed using N‐terminal sequencing and electrospray ionization mass spectrometry (ESI‐MS). Edman degradation of the excised p65 and p51 bands yielded the same N‐terminal sequence [I‐D‐I‐L‐A‐P‐Q], indicating that they originated from the same precursor (Figure 2d). The molecular masses measured by ESI‐MS were 64,875 Da and 51,209 Da for p65 and p51, respectively, which is consistent with the cleavage site at the [NNAL↓LVFT] site between the polymerase and RNase H domains (Figure 2d).

To further assess heterodimeric formation, we generated a truncated RT construct carrying RT lacking the RNase H domain (RTΔH) and co‐expressed this form with full‐length RT (His‐TEV‐RT‐TEV‐MBP) in Sf9 cells. Purification via the N‐terminal His tag, which is present only in full‐length RT, co‐isolated both the p65 and p51 subunits. These were further purified by SEC, yielding a sample that contained both p65/p51 subunits (Figure 2e, second lane). These results demonstrate that M‐PMV RT undergoes viral protease‐mediated processing to generate p65 and p51 subunits, which likely form heterodimeric p65/p51 complexes.

To assess the structural stability of RT and RTΔH, we analyzed both forms using nano‐differential scanning fluorimetry (nanoDSF) combined with back‐reflection turbidimetric measurements. The melting temperature (*T*_*M*_) indicates a point at which 50% of the protein population is unfolded, whereas the temperature of the aggregation onset (*T*_Agg_) corresponds to the temperature at which detectable protein aggregation begins. Full‐length RT exhibited a slightly higher *T*_*M*_ (by ~1°C) but a lower *T*_Agg_ (by ~3°C) compared to RTΔH (Figure 2f). Notably, the reduced *T*_Agg_ observed for full‐length RT likely reflects the presence of aggregation‐prone species rather than the intrinsic stability of the native protein. This interpretation is supported by the unfolding and turbidity profiles shown in Figure S1, which indicate early aggregation preceding complete unfolding in the homodimeric RT.

### 2.3 Oligomerization of M‐PMV reverse transcriptase

**FIGURE 3:**
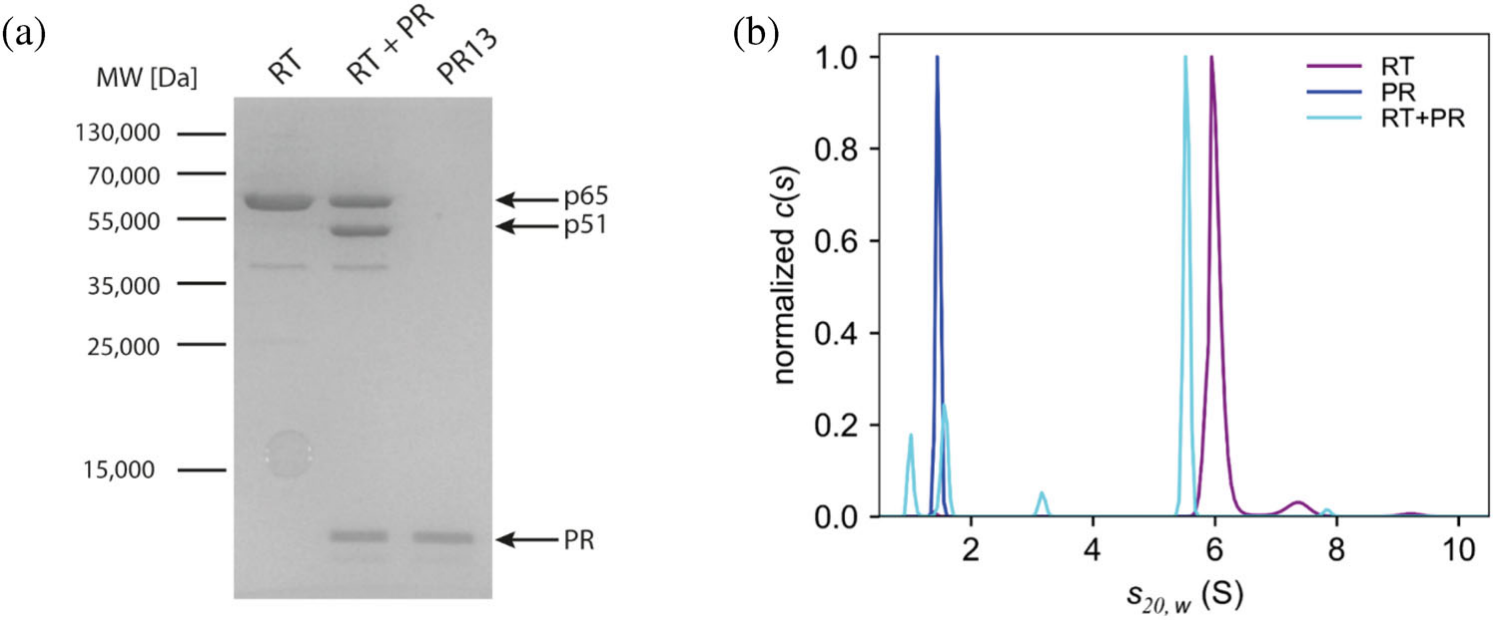
(a) SDS‐PAGE analysis of full‐length RT sample alone (RT), in the presence of M‐PMV PR13 (RT + PR), and M‐PMV PR13 alone (PR13). The full‐length RT lane shows a single band corresponding to p65 (~65 kDa). Upon proteolytic cleavage by PR13, an additional band corresponding to p51 (~51 kDa) appears, consistent with removal of the RNase H domain from one subunit. The protease itself migrates as a ~ 13 kDa band. (b) Sedimentation coefficient distribution profiles obtained by analytical ultracentrifugation. Uncleaved RT (purple) sedimented as a homodimer at ∼6.1 S. Upon cleavage by PR13, RT shifted to a heterodimeric form (p65/p51) and sedimented at ∼5.6 S (light blue). PR13 alone sedimented at ∼1.5 S (dark blue).

Our data thus far have shown that the proteolytic processing of full‐length RT generates two distinct subunits, p65 and p51. Because the co‐expressed p65 and p51 proteins co‐purified as a complex, we hypothesized they assemble into a heterodimeric RT. To test this directly, we analyzed the oligomeric state of both full‐length RT and RT cleaved by viral PR13 (Figure 3a) using sedimentation velocity analytical ultracentrifugation. Full‐length RT alone (purple) sedimented as a single, symmetric peak at 6.06 S (∼117 kDa), consistent with a homodimer (Figure 3b). Protease alone (dark blue) showed a sharp peak at 1.47 S (∼13 kDa), corresponding to its monomeric form. In the RT + PR sample (light blue), the RT peak shifted to 5.59 S (∼107 kDa), and the original ∼6.1 S homodimer peak was absent, indicating complete conversion. The other peaks observed in this sample corresponded to the added PR (∼1.6 S), cleaved RNase H domain (∼1.0 S), and probably also a residual amount of the cleaved MBP tag (∼3.2 S), visible also on the SDS‐PAGE gel at ∼40 kDa (Figure 3a). No free RT subunits were detected, confirming their complete dimeric assembly.

The molecular weights predicted from the measured sedimentation coefficients did not precisely match the theoretical molecular weights of either the 130 kDa homodimer or the 116 kDa heterodimer; however, their relative difference of ∼10 kDa points toward cleavage of a single RNase H domain only, not both. Also, no signals corresponding to monomeric RT (65 kDa) or RTΔH (51 kDa) were detected, thus both cleaved and uncleaved RTs must exist exclusively in dimeric form. The full‐length uncleaved RT forms a homodimer composed of two p65 subunits that sediment at ∼6.1 S. Upon protease cleavage, RT is converted into a heterodimer consisting of one p65 and one p51 subunit. This interpretation is supported by the apparent shift in the sedimentation coefficient distribution profile: cleaved RT displayed a single, distinct peak at ~5.6 S, indicating the formation of a homogeneous species. Together with the ESI‐MS data, these findings strongly support the existence of a stable p65/p51 heterodimer. Importantly, analysis of virion‐derived RT revealed two distinct bands corresponding to p65 and p51, confirming that protease‐mediated processing and heterodimer formation also occur during viral maturation.

### 2.4 Enzymatic activity of M‐PMV reverse transcriptase

**FIGURE 4:**
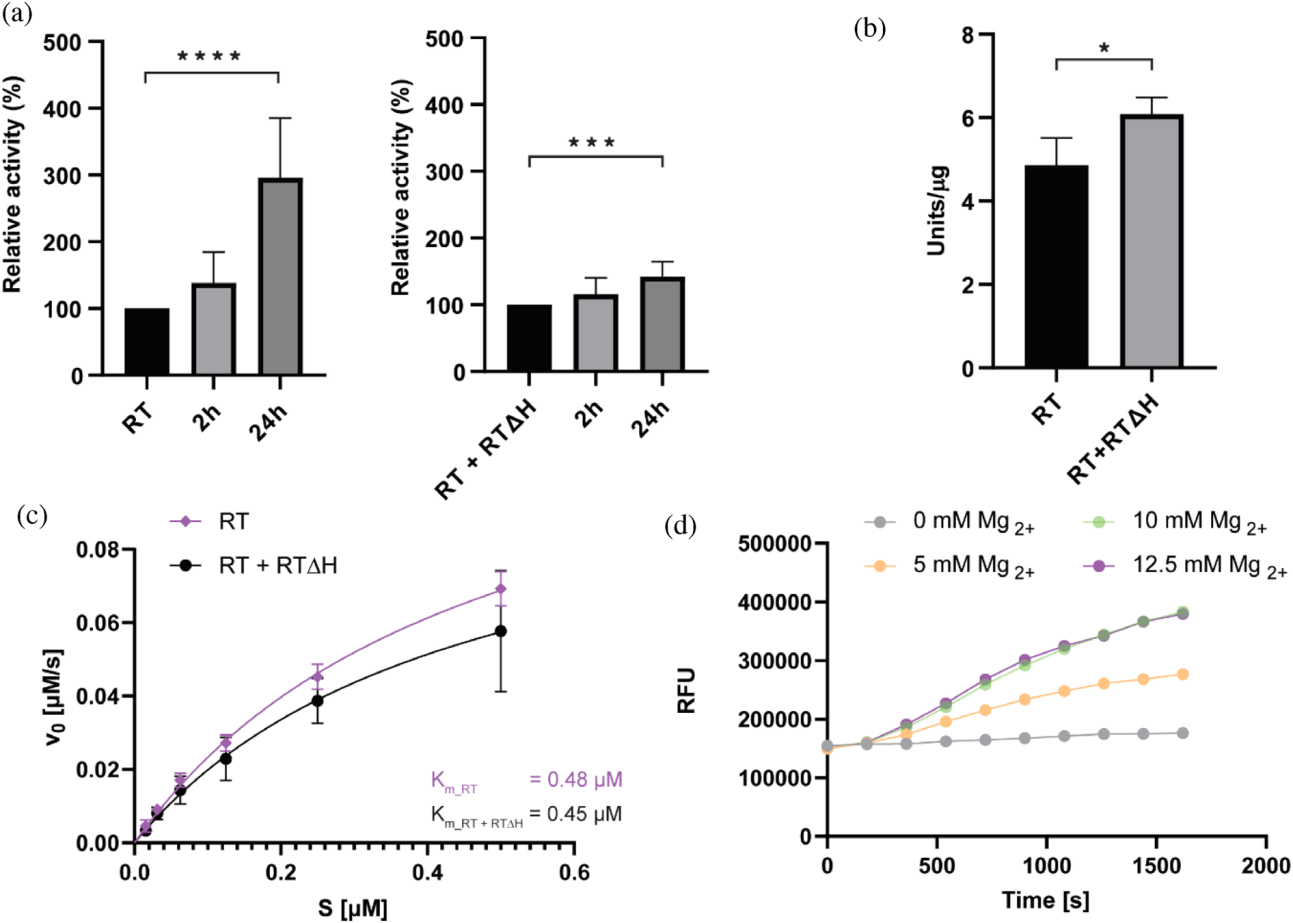
Enzymatic characterization of M‐PMV reverse transcriptase. (a) qPCR‐based analysis of polymerase activity of RT (left) and RT + RTΔH (right) after 2 and 24 h of incubation with M‐PMV protease. Statistical significance was determined using Welch's unpaired *t*‐test (*n* = 3, ***p < 0.001; ****p < 0.0001). (b) Polymerase activity of RT and RT + RTΔH was measured using a colorimetric assay. Statistical significance was determined using a standard unpaired *t*‐test (*n* = 3, *p < 0.05). (c) Michaelis–Menten analysis of RNase H activity showing fit to the Michaelis–Menten equation to calculate *K*_*M*_ and *V*_max_ values. (d) RNase H activity of M‐PMV RT at varying Mg^2+^ concentrations. Substrate cleavage was monitored as an increase in fluorescence over time.

The enzymatic activity of M‐PMV RT was initially assessed using a quantitative PCR (qPCR)‐based assay with a fixed amount of RNA template and SYTO‐9 detection, which allowed quantification of cDNA synthesis. All reactions were performed with equal amounts of RNA, with a non‐template control and in‐house murine RT as a positive control. To test the direct impact of proteolytic maturation on RT polymerase activity, full‐length RT was incubated with M‐PMV PR13, and the reaction was monitored for 24 h. Proteolytic removal of one RNase H domain significantly increased the reverse transcriptase polymerase activity. The cleaved RT showed enhanced reverse transcription activity, with 38.2 ± 18.9% of the initial signal after 2 h and 295.9 ± 28.3% after 24 h, compared to the non‐cleaved form, as calculated by the relative quantification method (Figure 4a, left panel). No RT activity was detected in the PR13 protease sample, with Cq values comparable to the non‐template control, indicating that neither the protease itself nor any proteins potentially present in the sample contributed to reverse transcription.

In contrast, the addition of PR13 to the RT + RTΔH complex modestly increased RT polymerase activity. This construct retained six additional residues at the N‐terminus and nine at the C‐terminus, following tag removal during purification. These residues were likely cleaved by the M‐PMV protease, which enhanced RT's activity. The relative activity of the RT + RTΔH complex increased by approximately 42.0 ± 7.1% after 24 h of incubation with PR13 compared to that of the untreated control (Figure 4a, right panel).

The polymerase activity of recombinant RT and RT + RTΔH was further assessed using a colorimetric assay based on DNA synthesis on a poly(A)·(dT)₁₅ template/primer by incorporating digoxigenin‐ and biotin‐labeled nucleotides. The full‐length RT exhibited an activity of 4.9 ± 0.8 U μg^−1^, whereas RT + RTΔH showed slightly higher activity at 6.1 ± 0.6 U μg^−1^ (Figure 4b). For comparison, the HIV‐1 RT provided with the assay kit displayed an activity of >5 U μg^−1^. These results confirm that both M‐PMV RT variants are catalytically active, with RT + RTΔH exhibiting moderately increased activity compared to the full‐length enzyme.

In addition to polymerase activity, we also measured RNase H activity. As the full‐length RT homodimer contains two RNase H domains, whereas proteolytic maturation yields a heterodimer with only one, we investigated whether the loss of one RNase H domain affected endoribonuclease activity. Michaelis–Menten analysis of RNase H activity using an 18‐nt RNA/DNA duplex revealed a *K*_*M*_ of 0.48 μM (95% CI: 0.37–0.63 μM) and *V*_max_ of 0.135 μM·s^−1^ (95% CI: 0.117–0.159) for RT (Figure 4c). RT + RTΔH yielded a similar *K*_*M*_ estimate of 0.45 μM (95% CI: 0.23–1.11) with a *V*_max_ of 0.109 μM·s^−1^ (95% CI: 0.076–0.200). The RT + RTΔH enzyme was assayed for Mg^2+^ dependence, showing minimal activity in the absence of Mg^2+^ and a dose‐dependent increase with an optimum at 10–12.5 mM concentration (Figure 4d).

Together, these results demonstrate that protease‐mediated maturation of M‐PMV RT enhances reverse transcription activity, whereas both full‐length and truncated enzymes retain robust polymerase and RNase H activities.

### 2.5 Effect of cleavage‐site mutations between the polymerase and RNase H domains on RT processing, activity, and M‐PMV infectivity

To investigate whether the proteolytic processing of RT could be impaired, we introduced mutations into the cleavage site between the polymerase and RNase H domains. As retroviral proteases preferentially recognize substrates containing hydrophobic residues at the scissile bond (represented in M‐PMV by two leucines, L447 and L448, Figure 2d), we designed six substitutions targeting only the P1 and P1' positions. The mutations L447I and L447P were selected based on previous observations that HIV‐1 protease disfavors β‐branched amino acids and proline immediately upstream of the cleavage site (Tözsér, 2010). Substitutions L447Q, L447R, and L448T introduced polar residues, which are generally unfavorable for protease recognition. Finally, L448G was designed to introduce a small residue at P1', as proteases generally avoid small side chains at positions directly adjacent to the cleavage site. All mutations were introduced into the full‐length proviral pSARM construct containing the complete M‐PMV genome, as well as into the pSARM‐GFP vector, which lacks envelope proteins but expresses GFP enabling quantitative assessment of infectivity by flow cytometry.

**FIGURE 5:**
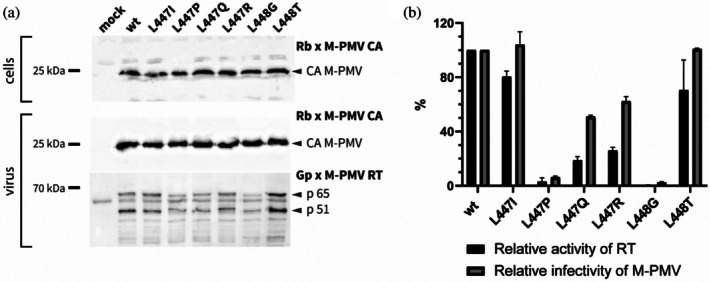
The effects of polymerase–RNase H cleavage‐site mutations on RT processing, enzymatic activity, and M‐PMV infectivity. (a) Western blot analysis of M‐PMV capsid (CA) and reverse transcriptase (RT) in HEK293 producer cells and purified virions containing wt RT or RT variants carrying a series of amino‐acid substitutions of L447 and L448 in the polymerase—RNase H cleavage site. The upper and middle panels show the levels of M‐PMV capsid protein (CA) in producer cell lysates and corresponding viral particles, detected using a rabbit anti‐M‐PMV CA antibody (Rb × M‐PMV CA). The lower panel shows immunodetection of RT in virions using a guinea‐pig anti‐RT antibody (Gp × M‐PMV RT). The presence of two RT subunits, p65 and p51, produced by RT processing, is indicated. (b) Relative RT enzymatic activity (black bars) and M‐PMV infectivity (gray bars), expressed as percentages of wt, were assessed. The amounts of released mutant viruses were normalized by ELISA based on M‐PMV capsid protein levels. RT activity and M‐PMV infectivity measurements were obtained from two independent biological replicates. Data are presented as mean ± SD.

Western blot analysis of M‐PMV capsid protein (CA) levels in HEK 293 cell lysates and corresponding virions demonstrated comparable CA abundance across all variants (Figure 5a, upper and middle panels), indicating that neither viral polyproteins nor particle assembly and release were substantially affected by any of the introduced mutations. Analysis of RT processing revealed that none of the mutations fully blocked protease‐mediated cleavage (Figure 5a, lower panel). The L447I, L447Q, and L448T mutants exhibited processing patterns similar to the wild‐type (wt) RT. In contrast, the L447P and L447R variants exhibited a shifted p51 band, suggesting that cleavage at the canonical site was impaired and that alternative cleavage occurred at a nearby position. For the L448G mutant, the p65 precursor was barely detectable, with p51 predominating, indicating altered processing.

Functional assessment of RT activity of released mutant viruses normalized by ELISA showed a pronounced reduction for nearly all mutants except L447I and L448T (Figure 5b). The most severe defect was observed in the L448G mutant, which retained <1% of wt RT activity. Measurements of relative M‐PMV infectivity correlated closely with the RT activity data: L447I and L448T displayed minimal reductions, whereas all other mutants, particularly L447P and L448G, exhibited near‐complete loss of infectivity.

## 3 DISCUSSION

Retroviral reverse transcriptases exhibit notable diversity in their oligomeric states and structural organization. Among the retroviral genera, HIV‐1 RT is the most extensively studied and is well‐known for its heterodimeric structure consisting of p66 and p51 subunits. However, the oligomeric states and maturation mechanisms of RT from other genera remain poorly characterized. Betaretroviruses, such as Mason‐Pfizer monkey virus, are considered to possess monomeric RTs, largely because of limited structural and biochemical data. Understanding the structure and maturation of M‐PMV RT is important not only for basic virology but also for expanding the scope of comparative retrovirology. In this study, we aimed to bridge this gap by providing detailed structural and biochemical characterizations of M‐PMV RT, focusing on its oligomeric state, proteolytic processing, and enzymatic function.

We successfully expressed full‐length recombinant M‐PMV RT using the baculovirus expression system in Sf9 insect cells (Figure 2a, lower panel). Expression attempts in *E. coli*, even with codon optimization, failed due to extensive degradation, suggesting that M‐PMV RT may require eukaryotic chaperones or folding machinery for proper stability. This contrasts with HIV‐1 RT, where both p66 and p51 subunits are efficiently produced and reconstituted in *E. coli* (Müller et al., 1989; Stahlhut et al., 1994). Although our initial aim was to express full‐length RT alone, based on the detection of two distinct RT‐derived proteins in M‐PMV virions, we performed its co‐expression with the RTΔH variant (Figure 2e), during which we observed markedly improved stability of the truncated protein. When expressed individually, RTΔH was highly prone to degradation; however, co‐expression with full‐length RT led to protein stabilization. Notably, the RTΔH variant carried no affinity tag, yet it co‐purified with His‐tagged full‐length RT using IMAC, suggesting that the two proteins form a stable complex during expression. Moreover, after tag cleavage, full‐length RT and RTΔH were readily separated from the MBP tag by size‐exclusion chromatography, despite the slight size difference between RTΔH (~51 kDa) and MBP (~40 kDa). This unexpectedly efficient separation further suggests that RTΔH is incorporated into a complex rather than existing as a free monomer.

These data correlate nicely with the detection of two, p65/p51 distinct RT‐derived bands in M‐PMV viral particles (Figure 2b), suggesting the presence of a heterodimer. To our knowledge, no previous study has directly demonstrated heterodimer formation in betaretroviruses. Although Křížová et al. (Křízová et al., 2012) mentioned a possibility of heterodimeric RT, their assumptions were based on the presence of a 50 kDa construct reactive to G‐patch antibody and not on the reverse transcriptase itself. However, our ESI‐MS data showed that neither p65 nor p51 retains the N‐terminal G‐patch domain after proteolytic cleavage in vitro. Notably, these results are derived from recombinant protein processing, and the presence or absence of G‐patch in virion‐derived RT remains uncertain and difficult to compare directly to HIV‐1 RT, which lacks this domain entirely. Detection of two RT subunits in M‐PMV virions provided direct evidence of proteolytic processing during viral maturation.

To further analyze whether M‐PMV RT undergoes a protease‐mediated maturation process that parallels the heterodimer formation seen in HIV‐1 RT, we performed in vitro processing of RT by the viral protease. Proteolytic cleavage using recombinant M‐PMV protease (PR13) generated two major products: a ~65 kDa full‐length subunit (p65) and a ~51 kDa truncated subunit (p51) (Figure 2c) sharing the same N‐terminal sequence (Figure 2d), resembling the p66/p51 profile seen in HIV‐1 RT (Schulze et al., 1991). Even after 24 h, cleavage was incomplete, indicating that a conformational constraint may prevent M‐PMV protease from processing the remaining RNase H domain. Additionally, the cleavage site between polymerase and RNase H domains [NNAL↓LVFT] revealed by ESI‐MS aligns with known retroviral protease specificity, which typically targets two types of sites: one featuring a proline immediately upstream of the scissile bond, and another characterized by two large hydrophobic residues flanking the cleavage site (Tözsér, 2010). In the case of M‐PMV RT, the latter motif applies, suggesting that the protease recognizes a conserved structural motif shared with other retroviral substrates.

Sedimentation velocity analysis provides direct biophysical evidence for the structural transition of M‐PMV RT upon proteolytic cleavage. Analytical ultracentrifugation revealed a shift from a homodimer to a heterodimer following cleavage with M‐PMV protease, with no evidence of free monomers in the post‐cleavage sample (Figure 3b). While the molecular weights predicted from the measured sedimentation coefficients were slightly underestimated compared to the theoretical masses, SDS‐PAGE analysis of the same samples (Figure 3a) confirmed the presence of both cleaved and uncleaved forms in roughly equal proportions. These results support a model in which proteolytic processing converts a homodimeric precursor into a stable heterodimer, with one subunit lacking the RNase H domain. Notably, while a study by Müller et al., 1989 indicated incomplete dimerization of p66, the analytical ultracentrifugation data presented here showed complete dimerization of homodimeric RT, with no detectable monomeric subunits. This difference may reflect the expression system used, as M‐PMV RT was produced in insect cells, whereas early studies of HIV‐1 RT were performed using *E. coli*.

Proteolytic maturation increases the structural stability of M‐PMV RT. Comparison of the homodimeric RT and the heterodimeric RT + RTΔH using nano‐differential scanning fluorimetry combined with turbidity assays revealed differences in their thermal behavior. The heterodimer displayed a higher aggregation temperature relative to the RT homodimer, indicating increased resistance to aggregation (Figure 2f). Although the apparent melting temperature of the homodimeric RT was slightly higher, the unfolding curves showed that this signal was dominated by the unfolding of aggregated species rather than the native protein itself, underscoring that aggregation precedes unfolding in the homodimeric form.

Proteolytic cleavage significantly enhances the enzymatic activity of M‐PMV RT. Activity assays revealed an approximately 3‐fold increase in the activity of the cleaved RT compared to the uncleaved form after 24 h (Figure 4a, left panel). The RT + RTΔH variant also showed an increase, although to a lesser extent (Figure 4a, right panel). One possible explanation is the presence of additional residues at the N‐ and C‐termini that remained after tag removal during purification and reduced activity upon cleavage by M‐PMV protease. A more likely explanation is that the recombinantly prepared heterodimer does not strictly consist of 50% full‐length enzyme and 50% RTΔH; even a small proportion of homodimer could affect the measured activity. Previous studies on HIV‐1 RT reported 2.5‐fold (Müller et al., 1989) and 4‐fold (Stahlhut et al., 1994) increases in activity for the heterodimeric p66/p51 form compared to the homodimeric p66, which is in line with our observations. Comparison of enzyme activities using the colorimetric assay revealed that RTΔH was ~25% more active than full‐length RT (Figure 4b). However, this assay uses a different substrate and is primarily intended for quantifying RT concentration in samples (e.g., virions), so variability in the results is expected.

To further characterize enzymatic activities, we performed an RNase H fluorescence‐based assay. Kinetic analysis showed that M‐PMV RT cleaves RNA/DNA duplex substrate with a *K*_*m*_ of 0.48 μM, closely matching the value obtained for RT + RTΔH (0.45 μM) (Figure 4c). These similar affinities may indicate that only one RNase H domain is catalytically active in both the heterodimer and homodimer. Reported *K*_*m*_ values for HIV‐1 RNase H vary considerably, ranging from ~25 ± 3 nM under similar conditions using a fluorescence‐based assay (Parniak et al., 2003) to values in the micromolar range in studies using a gel‐based assay (Sevilya et al., 2003). M‐PMV RT also showed a strong dependence on Mg^2+^, with negligible activity in the absence of Mg^2+^ and maximal activity at 10–12.5 mM Mg^2+^ (Figure 4d).

The accurate proteolytic release of RT from the Gag‐Pro‐Pol polyprotein precursor and the correct cleavage at the polymerase–RNase H junction are both required for the functionality of RT derived from the virus. Our results demonstrate that mutational disruption of the polymerase–RNase H cleavage site in M‐PMV RT does not prevent protease‐mediated processing. This finding is consistent with the previous report on HIV‐1 RT cleavage‐site mutants (Abram & Parniak, 2005). In that study, extensive substitutions throughout the polymerase–RNase H cleavage junction also failed to block processing. Rather than accumulating as p66 homodimers, these substitutions led to populations of virions containing unstable RT, dominated by p51 or degraded products. Consistent with these findings, none of our P1 or P1' mutations fully blocked the polymerase–RNase H cleavage (Figure 5a, lower panel). While substitutions L447I, L447Q, and L448T preserved wild‐type‐like cleavage patterns, the altered electrophoretic mobility of the p51 subunit in the L447P and L447R mutants indicates that disruption of the canonical scissile bond can redirect proteolysis to alternative nearby cleavage sites. In the case of the L448G mutant, this substitution appears to substantially disturb proper RT folding, likely exposing both cleavage sites on the p65 subunits within the homodimeric precursor. Although RT‐derived products with molecular weights similar to p65 and p51 were still detected, most cleavage‐site mutants exhibited severe reductions in enzymatic activity and viral infectivity (Figure 5b). These results suggest that proper enzyme activity requires precise folding in conjunction with accurate, site‐specific processing at the polymerase–RNase H junction.

Sequence similarity alone does not predict the oligomeric architecture of retroviral reverse transcriptases. Remarkably, M‐PMV RT shares over 62% sequence identity with monomeric MMTV RT in fingers and palm subdomains yet adopts a dimeric structure more similar to HIV‐1 RT, with which it shares only ~32% identity (Figure 1a). This disconnect suggests that heterodimeric architecture may have evolved independently in different retroviral lineages, driven by similar functional pressures such as the need for asymmetric active sites or subunit stability. This structural similarity across distant viruses points to shared functional constraints beyond phylogenetic relationships.

One might ask why structure prediction tools such as AlphaFold were not used to resolve the oligomeric architecture of M‐PMV RT? In fact, we did attempt in silico modeling using AlphaFold; however, viral proteins remain underrepresented in the training data, and the algorithm tended to return conformations resembling known structures, most notably HIV‐1 RT. One of the predicted models of the M‐PMV RT heterodimer was nearly identical to the p66/p51 heterodimer of HIV‐1 RT. Moreover, depending on the random seed, AlphaFold produced multiple divergent models, indicating low prediction confidence. These inconsistencies illustrate the limitations of current AI‐based prediction tools when applied to structurally uncharacterized viral proteins and further underscore the need for high‐resolution structural methods, such as X‐ray crystallography or cryo‐EM, to determine the authentic architecture of M‐PMV RT.

Taken together, our findings establish that M‐PMV reverse transcriptase undergoes proteolytic maturation to form a stable, enzymatically active heterodimer. This mechanism was previously considered restricted only to lentiviral and alpharetroviral RTs. This contrasts with the prevailing view that betaretroviral RTs, such as MMTV, exist exclusively as monomers. Our results challenge long‐held assumptions about RT architecture and suggest that heterodimeric maturation may be a broader, conserved feature among retroviruses. High‐resolution structural studies of M‐PMV RT will be essential to define its architecture and uncover lineage‐specific features that could advance our understanding of retroviral diversity and evolution.

## 4 CONCLUSION

Our study provides the first biochemical evidence that the reverse transcriptase of Mason‐Pfizer monkey virus undergoes proteolytic maturation to form a functional heterodimer, similar to the well‐characterized HIV‐1 RT. We demonstrate that the cleaved heterodimeric form is enzymatically more active and that its formation depends on specific conditions, including protease activity at physiological temperature. Mass spectrometry, N‐terminal sequencing, and analytical ultracentrifugation collectively support a model in which a homodimer is processed into a stable heterodimer. Furthermore, we have shown that precise folding combined with correct, site‐specific processing is required for proper enzyme function. These results broaden our understanding of reverse transcriptase maturation across retroviral genera and offer new insights into the evolutionary relationships between distantly related RTs. By revealing unexpected structural parallels, this study contributes to a more complete view of retroviral diversity and may support the development of alternative RT enzymes for molecular biology applications.

## 5 MATERIALS AND METHODS

### 5.1 Amino acid sequence alignment

To compare the reverse transcriptases from different retroviral genera, amino acid sequences corresponding to the polymerase domain (fingers and palm subdomains) were retrieved from the UniProt database (Consortium 2024). Specifically, the following entries were used: HIV‐1 (P04585, residues 1–235), MMTV (P11283, residues 1–234), BLV (P25059, residues 1–233), ALV (Q7SQ98, residues 1–189), and MLV (P03356, residues 1–189). The M‐PMV RT sequence was obtained from the pSARM vector containing the M‐PMV genome (Kohoutová et al., 2009). All sequences were aligned using the MAFFT algorithm via Jalview v2.11.2.5 with default parameters (MAFFT [web service]), and pairwise sequence identities were calculated within Jalview (Troshin et al., 2011; Troshin et al., 2018).

### 5.2 Preparation of expression vectors for in vitro protein production

The expression vectors encoding full‐length RT (6 × His‐TEV‐RT‐TEV‐MBP) and RT variant lacking the RNase H domain (RTΔH) were constructed using the Bac‐to‐Bac™ baculovirus expression system (Invitrogen, USA). The RT coding sequence for both full‐length RT and RTΔH was cloned from the pSARM vector into the pFastBac HT vector. Used primers: 5′ Xho His TEV (AAACTCGAGATGTCGTACTACCATCACCATCACC), 3′ RTstop KpnI (TTTGGTACCTTAAGCCACGATTTTAGTTGCCAAGTCAGCCCGTTGGTTGCCTTGAGCTATGGG) and FOR RT dH pACM4 NdeI (TTTTCATATGATTGACATACTTGCAC), REV RT dH pACMV4 KpnI (TTTGGTACCTCATAAGGCATTGTTT) for RTΔH. The pFastBac vectors containing RT sequences were transformed into *Escherichia coli* DH10α. The isolated bacmid DNA was then transfected into Sf9 insect cells to generate an infectious viral stock. To prepare M‐PMV PR13 protease, the pSIT vector (Veverka et al., 2003) was used. All constructs were verified by Sanger sequencing.

### 5.3 Protein production and purification

- Sf9 cells were kept at 27°C in Sf‐900 II medium (Invitrogen, USA). For protein expression, two strategies were used: (i) expression of 6 × His‐TEV‐RT‐TEV‐MBP alone, and (ii) co‐expression of full‐length RT with RTΔH to mimic heterodimer formation. In both cases, Sf9 cells were transfected with bacmid DNA, and baculovirus‐containing supernatants were harvested 5 days post‐transfection. The baculoviral stock was subsequently amplified to achieve optimal infectivity. Suspensions of Sf9 cells (3 × 10^6^ cells ml^−1^) were infected with the amplified baculovirus at a 1:100 v/v ratio. After 48 h, cells were harvested by centrifugation (3000×*g*, 15 min).
  The harvested cell pellets were resuspended in lysis buffer (25 mM Na₂HPO₄, pH 7.4; 500 mM NaCl; 250 mM trehalose; 2 mM MgCl₂; 20 mM imidazole; 2 mM β‐mercaptoethanol) and homogenized using a One Shot Cell Disruptor (Constant Systems, United Kingdom) at 1.0 kbar. The lysates were clarified by centrifugation at 50,000×*g* for 30 min, and the supernatants were loaded onto a HisTrap™ HP column (Cytiva, USA) pre‐equilibrated with the same lysis buffer. For the full‐length RT sample, the column was additionally washed with high‐salt buffer (25 mM Na₂HPO₄, pH 7.4; 1 M NaCl; 0.5 M urea; 250 mM trehalose; 2 mM MgCl₂; 20 mM imidazole; 2 mM β‐mercaptoethanol) prior to elution. In both cases, the proteins were eluted using a 30–500 mM imidazole gradient.
  To remove the 6 × His‐TEV and TEV‐MBP tags, TEV protease was added at a 1:20 w/w ratio, and the eluate was dialyzed overnight at 4°C against 2 L of dialysis buffer (25 mM HEPES, pH 7.4; 200 mM NaCl; 50 mM trehalose; 2 mM MgCl₂; 2 mM β‐mercaptoethanol). Following dialysis, samples were centrifuged at 20,000×*g* for 20 min, concentrated, and subjected to size‐exclusion chromatography (HiLoad 26/600 Superdex 200 pg., Cytiva, USA) in SEC buffer (25 mM Na‐HEPES, pH 7.4; 200 mM NaCl; 25 mM trehalose; 2 mM MgCl₂; 5% glycerol; 1 mM TCEP). Fractions containing purified RT were pooled, concentrated, and stored at −80°C.
- The vector encoding the active M‐PMV PR13 protease was expressed in *E. coli* BL21 (DE3) in LB medium supplemented with 100 μg ml^−1^ ampicillin. Protein expression was induced with 1 mM IPTG when cultures reached an OD_600_ of 0.9–1.1, followed by a 2‐h expression at 37°C. Cells were harvested by centrifugation, and PR13 was isolated from inclusion bodies similarly to the previously described method (Rumlová et al., 2014). The cell pellet was resuspended in buffer A (50 mM Tris, pH 8; 50 mM NaCl; 1 mM EDTA), followed by two washes with buffer B (50 mM Tris, pH 8; 1 M NaCl; 1 mM EDTA) and a final wash with buffer A. After each resuspension, samples were sonicated and centrifuged (10,000×*g*, 10 min). The final pellet was solubilized in 2 mL of 67% (v/v) acetic acid in aqueous solution at room temperature for 60 min, followed by sonication. The solution was then slowly dripped into 10 mL of cold water. The mixture was dialyzed against water for a maximum of 120 min, and then overnight against dialysis buffer (50 mM phosphate, pH 5.8; 1 mM EDTA; 0.05% β‐mercaptoethanol). The solution was filtered through a 0.22 μm filter, concentrated, and subjected to size‐exclusion chromatography using a HiLoad 26/600 Superdex 200 pg. (Cytiva, USA) column equilibrated with the same buffer. Fractions containing purified PR13 were pooled, concentrated, and stored at −80°C.

### 5.4 Proteolytic cleavage

Recombinant M‐PMV RT (final concentration 5.5 μM) was incubated with M‐PMV PR13 protease in cleavage buffer (25 mM Na‐HEPES, pH 7.4; 200 mM NaCl; 25 mM trehalose; 2 mM MgCl₂; 2 mM β‐mercaptoethanol) at a 1:1 molar ratio. Reactions were incubated at 37°C, 20°C, or 4°C for 30 min, 1 h, 2 h, 4 h, or 24 h. Proteolytic cleavage products were analyzed by SDS‐PAGE.

### 5.5 Thermal unfolding assay

RT and RTΔH were diluted to a concentration of 0.5 mg ml^−1^. Thermal unfolding parameters were then determined using a combination of nano‐differential scanning fluorimetry (nanoDSF) and turbidimetry using Prometheus Panta (Nanotemper, Germany). Protein solutions were heated at 1.2°C per minute from 25 to 95°C. Intrinsic fluorescence (λex = 280 nm, λem = 330 and 350 nm) and turbidity were measured throughout the process. Results were analyzed using the Panta Analysis software (Nanotemper, Germany). Melting temperature *T*_*M*_ represents the inflection point of the 330/350 fluorescence ratio curve whereas *T*_Agg_ represents the onset temperature of turbidity increase.

### 5.6 Protein sequencing

Purified reverse transcriptase was incubated with the M‐PMV PR13 protease overnight at 37°C in the cleavage buffer described in the proteolytic cleavage. Cleavage products were separated on SDS‐PAGE and electroblotted onto a PVDF membrane. N‐terminal amino acid sequences were determined by Edman degradation using the Procise Protein Sequencing System (Applied Biosystems, 491 HT or 494 cLC Protein Sequencer, USA). In this method, the free amino group of the N‐terminal amino acid reacts specifically with phenylisothiocyanate. The derivatized amino acid is then selectively cleaved from the polypeptide and converted into a stable phenylthiohydantoin (PTH) derivative, while the rest of the peptide remains intact. Each cycle removes one N‐terminal residue. The resulting PTH‐amino acids are sequentially analyzed by reversed‐phase HPLC, allowing determination of the N‐terminal amino acid sequence of the protein or peptide.

### 5.7 ESI MS

Samples including full‐length RT, cleaved RT (RT + PR13), and PR13 protease were diluted with 0.1% formic acid to a 0.2 mg ml^−1^ concentration. 10 μL of the sample was injected onto a desalting column (MassPREP desalting, Waters, USA) and desalted by fast gradient (4 min) of acetonitrile in water with 0.1% formic acid. The separation was carried out by an LC system (I‐class, Waters, USA) coupled online to a mass spectrometer (Synapt G2, Waters, USA) to acquire the mass of the protein by electrospray ionization. The raw spectrum was subtracted and deconvoluted (MaxEnt 1, Waters, USA) to produce the final spectrum.

### 5.8 Analytical ultracentrifugation

Sedimentation analysis, used to monitor the oligomeric state and associative behavior of biomacromolecules (Bláha et al., 2022; Skořepa et al., 2020), was performed on the analytical ultracentrifuge Optima AUC (Beckman Coulter, USA) using an An50‐Ti rotor and double‐sector cells equipped with 12 mm Epon centerpieces (Beckman Coulter, USA). Samples in 25 mM HEPES, pH 7.2; 200 mM NaCl; 2 mM MgCl_2_; 1 mM TCEP buffer were analyzed at 20°C and 42,000 rpm. The sedimentation velocity experiment was recorded as absorbance at 280 nm with 300 scans in 3‐min steps. Suitable initial sample concentrations were calculated using Lambert–Beer's law, assuming a 1.2 cm optical path length and 0.2–1.0 desired absorbance value (M‐PMV RT, 6.9 μM; M‐PMV PR, 6.9 μM; and RT + PR equimolar, 1:1 (v/v) mixture). Buffer density, protein partial specific volumes, and particle dimensions were estimated in Sednterp (Philo, 2023). Data were analyzed with Sedfit (Schuck, 2000) using the c(s) continuous sedimentation coefficient distribution model and visualized in GUSSI (Brautigam, 2015).

### 5.9 qPCR

Reverse transcription activity of cleaved RT was determined by RT‐qPCR using an artificial target SLA RNA (5′‐AGU UGU UAG UCU ACG UGG ACC GAC AAA GAC AGA UUC UUU GAG GGA GCU AAG CUC AAC GUA GUU CUA ACA GUU UUU U‐3′), where the recombinant protein served as the reverse transcriptase. The SLA RNA template was used at a final concentration of 100 nM, and the reverse transcriptase at a final concentration of 20 nM. The primers used were SLA primer (5′‐AGGGGGGGGGGTAAAAAACTGTTAGAACTA‐3′) for the initial reverse transcription to produce cDNA, and primers SLA qPCR F (5′‐TTGTTAGTCTACGTGGACCGA‐3′) and SLA qPCR R (5′‐AACTGTTAGAACTACGTT‐3′) to amplify the cDNA. qPCR reactions were conducted in 96‐well plates on a QuantStudio™ 5 Real‐Time PCR System (Applied Biosystems™, USA) using a Taq DNA polymerase–based master mix (Top‐Bio SYTO‐9 qPCR 2x Master Mix) under the following conditions: 15 min at 95°C; 40 cycles of 30 s at 95°C, 1 min at 60°C, and 2 min at 72°C. Measurement was performed three times in duplicates. RT activity corresponded to relative cDNA levels quantified using the comparative Cq method (ΔCq method) calculated as 2^^−ΔCq^. The activity of the non‐cleaved RT was set to 100%, and the activities of other samples were expressed relative to this value.

### 5.10 Colorimetric RT assay

Reverse transcriptase activity was quantified by colorimetric assay (Roche Applied Science, Cat. No. 11468120910) with exogenous poly(A)/oligo(dT) as the substrate/primer, according to the manufacturer's instructions. Reverse transcription was carried out for 1 h at 37°C. The reaction product was detected by measuring absorbance at 405 nm. All samples, including standards and controls, were assayed in three replicates. Negative controls without RT and blank wells containing only substrate were included to account for background signal.

### 5.11 Fluorescence‐based RNase H activity assay

RNase H activity was quantified using a fluorescence‐based assay in black 96‐well plates, with fluorescence measured on a SpectraMax iD5 microplate reader (Molecular Devices, USA). The substrate was a synthetic 18‐nt RNA/DNA hybrid composed of a 3′‐FAM‐labeled RNA strand (5′‐CACCAGCUCCGUAGUAGC‐[FAM]‐3′) annealed to a complementary 5′‐BHQ1‐labeled DNA strand (5′‐[BHQ1]‐CGATGATGCCTCGACCAC‐3′). Oligonucleotides were annealed in annealing buffer (50 mM HEPES, pH 8.0; 125 mM NaCl; 12.5 mM EDTA) by heating to 93°C for 3 min, followed by gradual cooling to room temperature. The resulting RNA/DNA substrate was aliquoted and stored at −80°C until use.

Reactions (100 μL) were carried out in FL assay buffer (50 mM HEPES, pH 7.5; 60 mM KCl; 10 mM MgCl₂; 2.5 mM DTT) with 1 nM recombinant M‐PMV RT (either RT or RT + RTΔH) and RNA/DNA hybrid at final concentrations ranging from 15 to 500 nM. Fluorescence was recorded every 4 min at 37°C for 1 h. Subsequently, an excess of enzyme was added to determine the maximum fluorescence (*F*_max_). Initial velocities (*v*_*₀*_) were obtained from the linear portion of the fluorescence increase (Δ*F*/Δ*t*) for each substrate concentration. The relative slopes were normalized to the total fluorescence change (Δ*F* = *F*_max_ − *F*_*o*_) and multiplied by the respective substrate concentration to yield apparent rates in concentration units (μM·s^−1^). These values were fitted to the Michaelis–Menten equation to estimate *K*_*m*_ and *V*_max_.

### 5.12 Preparation of expression vectors for M‐PMV virion production in mammalian cells

The proviral M‐PMV pSARM vector, as well as the pSARM‐EGFP construct, was used to introduce single‐point mutations into the cleavage site between the polymerase and RNase H domains. Based on the precisely defined sequence of the cleavage site [NNAL↓LVFT], six mutants were generated: four containing amino‐acid substitutions at the P1 position (L447I, L447P, L447Q, L447R) and two at the P1′ position (L448G, L448T) (Table S1). The corresponding mutagenic oligonucleotide primers used for construct generation are listed in Table S2. Single‐point mutations were introduced using the previously described EMILI method (Füzik et al., 2014). All constructs were verified by whole‐genome sequencing.

### 5.13 Cell cultures

HEK 293 cells were cultured in Dulbecco's modified Eagle medium (DMEM; Sigma, USA) supplemented with 10% fetal bovine serum (Sigma, USA) and 1% L‐glutamine (Sigma, USA) at 37°C in a humidified atmosphere containing 5% CO₂. One day prior to transfection, cells were seeded at a density of 3 × 10^5^ cells mL^−1^. The following day, cells were transfected with the M‐PMV proviral pSARM vector (wt or mutant constructs) using polyethylenimine at a DNA‐to‐transfection reagent ratio of 1:2 (w/v). After transfection, cells were incubated for an additional 48 h and subsequently used for downstream analyses.

### 5.14 M‐PMV virion production

At 48 h post‐transfection, virions released into the culture medium were harvested by ultracentrifugation through a 20% sucrose cushion at 39,000 rpm for 1.5 h at 10°C in an SW 40 Ti rotor (Beckman Coulter, USA). Viral pellets were resuspended in 60 μL of virion lysis buffer (50 mM Tris, pH 7.8; 80 mM KCl; 2.5 mM DTT; 0.75 mM EDTA; 0.5% Triton X‐100) and reverse transcriptase activity was quantified as described in the qPCR methods section. Samples were later combined in a 1:1 (v/v) ratio in loading buffer (0.4 M Tris; 4% SDS; 10% β‐mercaptoethanol; 24% glycerol; bromophenol blue) and analyzed using SDS‐PAGE. The procedure was performed as described previously (Dostálková et al., 2024).

### 5.15 Single‐round infectivity assay M‐PMV

The infectivity was determined as described earlier (Dostálková et al., 2020). Briefly, 48 h post‐transfection, the culture media from HEK 293 cells transfected with pSARM‐GFP (wt or mutant constructs) and pTMO vectors at a (w/v) ratio 1:1 were collected and filtered through a 0.45‐μm membrane filter. M‐PMV capsid protein content was determined by ELISA. The freshly seeded HEK 293 cells were infected with ELISA‐normalized amounts of M‐PMV particles and incubated for 48 h. The cells were fixed with 2% paraformaldehyde and transferred to a FACS tube. Quantification of GFP‐positive cells was performed using a BD FACS Aria III flow cytometer (BD Biosciences, USA).

### 5.16 Assessment of RT activity from released mutant viruses

Reverse transcriptase activity from the released virions was assessed using a previously described RT‐qPCR–based assay as detailed in this study. Viral particles were collected by centrifugation and resuspended in 60 μL of virion lysis buffer. From each lysate, 1 μL was used as input for the RT‐qPCR reaction. RT activity was quantified as described above. Measurements were performed using two independent biological replicates. To account for differences in viral particle production, the obtained RT activity values were normalized to the M‐PMV capsid protein levels determined by ELISA.

### 5.17 Flow cytometry

The infected cells were analyzed with a BD FACS Aria III flow cytometer (BD Biosciences, USA) with excitation at 488 nm and emission separated by a 530/30 band pass filter, as described earlier (Dostálková et al., 2020). The obtained data were analyzed with Diva 8 software (BD Biosciences, USA). Measurements were performed using two independent biological replicates.

## AUTHOR CONTRIBUTIONS

**Marina Kapisheva:** Conceptualization; writing – original draft; formal analysis; investigation; visualization. **Petra Junková:** Investigation; formal analysis. **Ondřej Vaněk:** Investigation; writing – review and editing. **Zuzana Jalovcová:** Investigation. **Ivana Křížová:** Methodology; investigation; formal analysis. **Alžběta Dostálková:** Methodology; writing – review and editing. **Michaela Rumlová:** Conceptualization; supervision; funding acquisition; project administration; writing – review and editing.

## Supporting information


**FIGURE S1.** Thermal unfolding profiles of RT + RTΔH (orange) and RT (blue). Ratio of fluorescence at 350 and 330 nm (top) was used to calculate the melting temperature (*T*_*M*_) indicated by dashed lines. Turbidity measurement (bottom) was utilized to obtain onset aggregation temperature (*T*_Agg_) shown as dashed lines.
**TABLE S1.** List of introduced mutations into the cleavage site between polymerase and RNase H domains.
**TABLE S2.** List of oligonucleotides utilized for mutagenesis of the cleavage site between polymerase and RNase H domains.

## Data Availability

The data that support the findings of this study are openly available in www.zenodo.org.
