# Supplementary material for: Protease‐mediated maturation of M‐PMV reverse transcriptase into a functional heterodimer
Source: Protein Sci. 2026 Jan 20;35(2):e70469. doi: 10.1002/pro.70469 (PMC12817477; doi:10.1002/pro.70469)
Supplement: Supplementary file 1 — FIGURE S1. Thermal unfolding profiles of RT + RTΔH (orange) and RT (blue). Ratio of fluorescence at 350 and 330 nm (top) was used to calculate the melting temperature (T M ) indicated by dashed lines. Turbidity measurement (bottom) was utilized to obtain onset aggregation temperature (T Agg) shown as dashed lines. TABLE S1. List of introduced mutations into the cleavage site between polymerase and RNase H domains. TABLE S2. List of oligonucleotides utilized for mutagenesis of the cleavage site between polymerase and RNase H domains. [file PRO-35-e70469-s001.pdf]

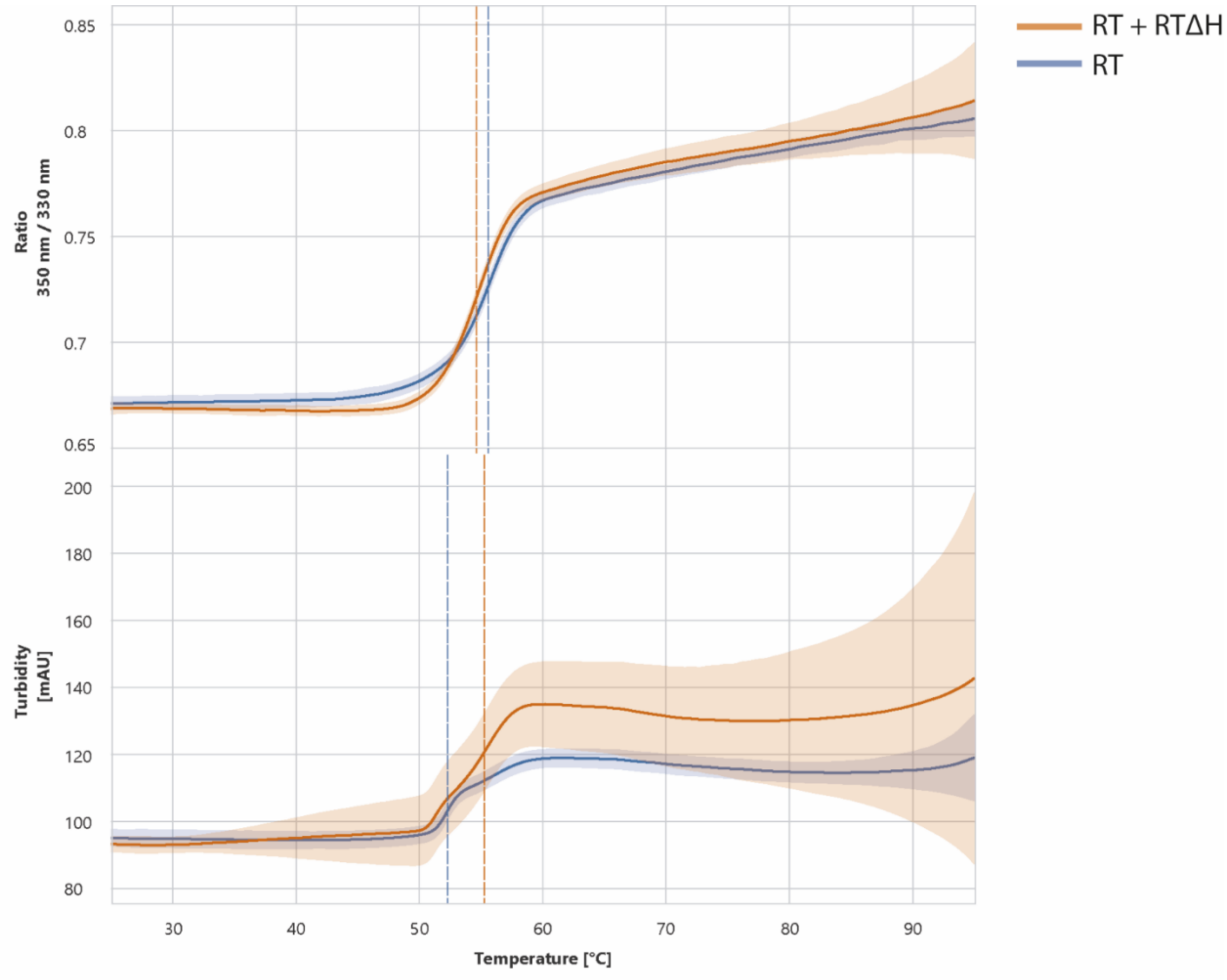

Figure S1. Thermal unfolding profiles of RT + RTΔH (orange) and RT (blue). Ratio of fluorescence at 350 nm and 330 nm (top) was used to calculate the melting temperature (T_M) indicated by dashed lines. Turbidity measurement (bottom) was utilized to obtain onset aggregation temperature (T_{Agg}) shown as dashed lines.

Table S1. List of introduced mutations into the cleavage site between polymerase and RNase H domains.

Abbreviation	Sequence (P3-P3')[a]	Substrate position	Classification
WT	NAL↓LVF	N/A [b]	N/A
L447I	NA***I***↓LVF	P1	Conservative
L447P	NA***P***↓LVF	P1	Radical
L447Q	NA***Q***↓LVF	P1	Radical
L447R	NA***R***↓LVF	P1	Radical
L448G	NAL↓***G***VF	P1'	Radical
L448T	NAL↓***T***VF	P1'	Radical

[a]Amino acid substitutions introduced at various positions flanking the scissile bond between polymerase and RNase H domains are in bold and italic. [b]N/A, not applicable.

Table S2. List of oligonucleotides utilized for mutagenesis of the cleavage site between polymerase and RNase H domains.

Oligonucleotide	Sequence
L447I pol F	GCCATATTAGTTTTTACTGATGGCTCTT
L447I pol R	AACTAATATGGCATTGTTTAAGGGTG
L447P pol F	GCCCCATTAGTTTTTACTGATGGCTCTT
L447P pol R	AACTAATGGGGCATTGTTTAAGGGTG
L447Q pol F	GCCCAATTAGTTTTTACTGATGGCTCTT
L447Q pol R	AACTAATTGGGCATTGTTTAAGGGTG
L447R pol F	GCCAGATTAGTTTTTACTGATGGCTCTT
L447R pol R	AACTAATCTGGCATTGTTTAAGGGTG
L448G pol F	GCCTTAGGAGTTTTTACTGATGGCTCTT
L448G pol R	AACTCCTAAGGCATTGTTTAAGGGTG
L448T pol F	GCCTTAACAGTTTTTACTGATGGCTCTT
L448T pol R	AACTGTTAAGGCATTGTTTAAGGGTG
